# Grape seed procyanidin extract against lung cancer: the role of microrna-106b, bioavailability, and bioactivity

**DOI:** 10.18632/oncotarget.24528

**Published:** 2018-02-16

**Authors:** Bingye Xue, Qing-Yi Lu, Larry Massie, Clifford Qualls, Jenny T. Mao

**Affiliations:** ^1^ Pulmonary, Critical Care, and Sleep Section, New Mexico Veterans Administration Health Care System, University of New Mexico, Biomedical Research Institute of New Mexico, Albuquerque, NM, USA; ^2^ UCLA Center for Human Nutrition, David Geffen School of Medicine at UCLA, Los Angeles, CA, USA; ^3^ Pathology and Clinical Laboratory Services, New Mexico Veterans Administration Health Care System, University of New Mexico, Albuquerque, NM, USA; ^4^ Biomedical Research Institute of New Mexico, New Mexico Veterans Administration Health Care System, University of New Mexico, Albuquerque, NM, USA

**Keywords:** oncomir, P21, CDKN1A, pharmacokinetics, pharmacodynamics

## Abstract

MiR-106b is an oncomir and a potential target for anti-cancer therapy. We hypothesize that grape seed procyanidin extract (GSE) exerts antineoplastic effects on lung cancer through modulations of miR-106b and its downstream target. We found that GSE significantly down-regulated miR-106b in a variety of lung neoplastic cells and increased cyclin-dependent kinase inhibitor 1A (*CDKN1A*) mRNA and protein (p21) levels. Transfection of miR-106b mimics reversed the up-regulations of *CDKN1A* mRNA and p21, abrogated the GSE induced anti-proliferative and anti-invasive properties in lung cancer cells. Oral gavage of leucoselect phytosome (LP), a standardized GSE to athymic nude mice down-regulated *MIR106B* mRNA and miR-106b expressions, and increased *CDKN1A* mRNA expression in tumor xenografts, correlating to significant reduction of tumor growth. To assess bioavailability, GSE and metabolites in plasma levels, between 60–90 minutes after gavage of LP were measured by LC/MS at treatment week 4 and 8. A novel bioactivity assay was also developed using lung homogenates from treated mice co-cultured with human lung cancer cells. LP-treated mouse lung homogenates significantly reduced proliferations of various lung cancer cells. Our findings reveal novel antineoplastic mechanisms by GSE, further define the pharmacokinetics and pharmacodynamics of LP, and support the continued investigation of LP against lung cancer.

## INTRODUCTION

Derived from seeds of grapes (*Vitis vinifera)*, GSE is high in procyanidins with strong antioxidant capabilities [1, 2]. GSE is mainly used as a health food supplement to improve cardiovascular health [3]. Preclinical studies have shown antineoplastic effects of GSE against a variety of cancers, including lung cancer [4–9]. We recently reported that modulations of oncogenic microRNA (miRNA) or oncomir miR-19a/b, contributed to the antineoplastic properties of GSE against non-small cell lung cancer (NSCLC) and bronchial premalignant cells [8]. MiRNAs are small noncoding RNA molecules with regulatory function and tissue specificity that can modulate multiple molecular targets across several mechanistic pathways. Through mRNA degradation and/or translational repression, miRNAs function as epigenetic modifiers and mediate post-transcriptional regulation of specific mRNAs [10]. Various miRNAs have been identified as oncomirs, based on their associations with cancer [11]. Oncomirs can mediate pro- or anti-tumor effects, are stable against degradation, and can be easily quantified in tissue samples and body fluids with simple assays like real time (q)PCR. Ample studies have shown that specific miRNA signatures in biospecimens had remarkable sensitivity and specificity in discriminating cancer patients from healthy subjects [12, 13]. These characteristics support the potential of oncomirs as therapeutic targets and surrogate endpoint biomarkers (SEBM) for lung cancer treatment and chemoprevention studies.

In this study, we found that GSE significantly down-regulated the expression of oncomir miR-106b in a variety of human lung cancer cell lines, including A549 (adenocarcinoma), H1299 (metastatic NSCLC), DMS114 (Small cell lung cancer, SCLC), and H23 (adenocarcinoma). Reduction of miR-106b correlated with decreased human lung cancer cell proliferations, as well as up-regulations of *CDKN1A* mRNA expressions, and the respective protein product p21, a predicted target of miR-106b (TargetScanHuman, http://www.targetscan.org/vert_61). Transfection with miR-106b mimic significantly reversed the anti-proliferative effects of GSE in lung cancer cells, and abrogated the GSE-induced up-regulation of *CDKN1A* and p21. In addition, transfection with miR-106b mimic significantly reversed the anti-invasive effects of GSE in A549 cells.

To facilitate future translation into clinical trials, we selected an inexpensive GSE preparation, leucoselect phytosome (LP), standardized to smaller size grape seed oligomeric procyanidins (OPC) and complexed with soy phospholipids into phytosomes to improve bioavailability, for our *in vivo* preclinical efficacy study [8]. Oral gavage of LP to athymic nude mice bearing A549 NSCLC xenografts significantly down-regulated the expressions of miR-106b and its precursor *MIR106B* mRNA, and increased *CDKN1A* mRNA expression in tumor xenografts, correlating to markedly reduced tumor growth.

To further define the bioavailability of LP, levels of GSE (procyanidins B1 and B2) and metabolites in plasma samples obtained 60–90 minutes after oral gavage were measured. The concentration of GSE that was associated with 50% *in vitro* tumor cell growth inhibition or cytotoxicity (IC_50_) based on MTT assay (45 μg/ml) [8], was much higher than the *in vivo* IC_50_ of the sum of plasma GSE and metabolites levels (0.875 μg/ml), obtained from the athymic nude mouse tumor xenograft model.

We also designed a novel fresh frozen lung homogenate co-culture with lung neoplastic cells as a surrogate model system to assess the bioactivity of orally administered LP in the lungs. Lung homogenates from LP-treated mice dose-dependently inhibited proliferations of a variety of lung cancer cell types. Our findings reveal novel anti-neoplastic mechanisms by GSE, demonstrate systemic bioavailability of LP and bioactivity of LP in the lungs, and support the further investigation of LP as an anti-neoplastic and chemopreventive agent for lung cancer.

## RESULTS

### GSE significantly down-regulated expressions of oncomir miR-106b, and mRNA of its precursor *MIR106B* gen*e* in lung neoplastic cells

**Figure 1:**
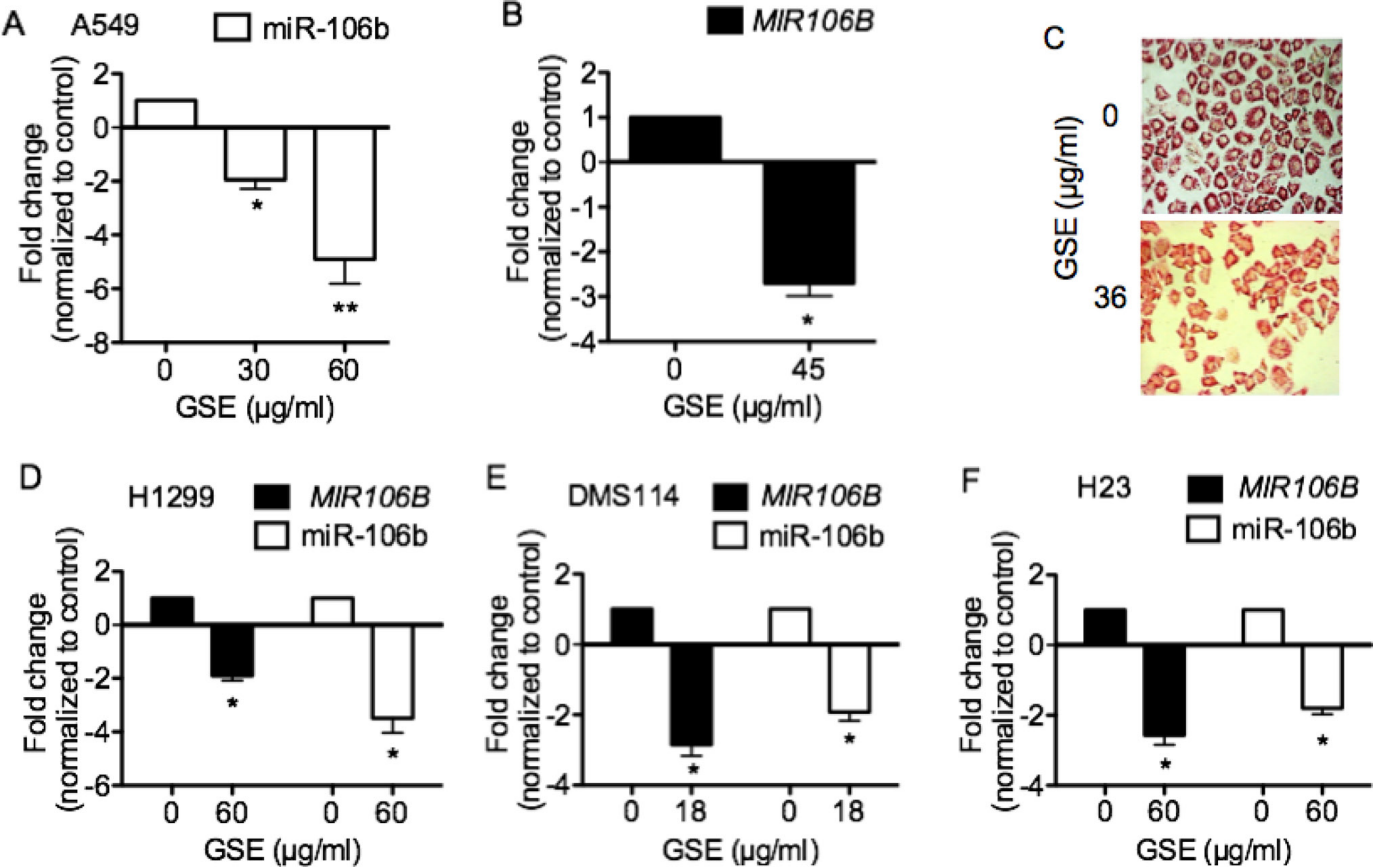
GSE significantly down-regulated oncomir miR-106b expression, and its precursor *MIR106B* mRNA expression in lung neoplastic cells Specific qPCR demonstrated down-regulation of (**A**) miR-106b, (**B**) *MIR106B* mRNA in A549 cells (*n* = 3). MiR-106b ISH assays further confirmed the down-regulation of these miRNA in A549 cells by GSE. (**C**) Representative photomicrograph of miR-106b specific ISH assay with fast red stain in conditioned A549 cells. Magnification: 400×. (**D**) GSE also significantly down-regulated the expression of miR-106b and MIR106B mRNA in (D) H1299 cells, (**E**) DMS114 cells and (**F**) H23 cells (*n* = 3). Columns, mean; bars, SD. ^*^P < 0.05; ^**^P < 0.01.

Specific qPCR demonstrated the dose-dependent, down-regulation of miR-106b (Figure 1A), its precursor *MIR106B* gene (Figure 1B) and further confirmation was obtained with miR-106b specific ISH assay in A549 cells (Figure 1C). GSE also down-regulated both miR-106b and *MIR106B* precursor in H1299 (Figure 1D), DMS114 (Figure 1E), and H23 cells (Figure 1F). GSE, however, did not down-regulate miR-106b in H520 cells (data not shown).

### GSE induced anti-proliferative effects in lung neoplastic cells via down-regulation of miR-106b, which was abrogated by transfection of miR-106b mimic

**Figure 2:**
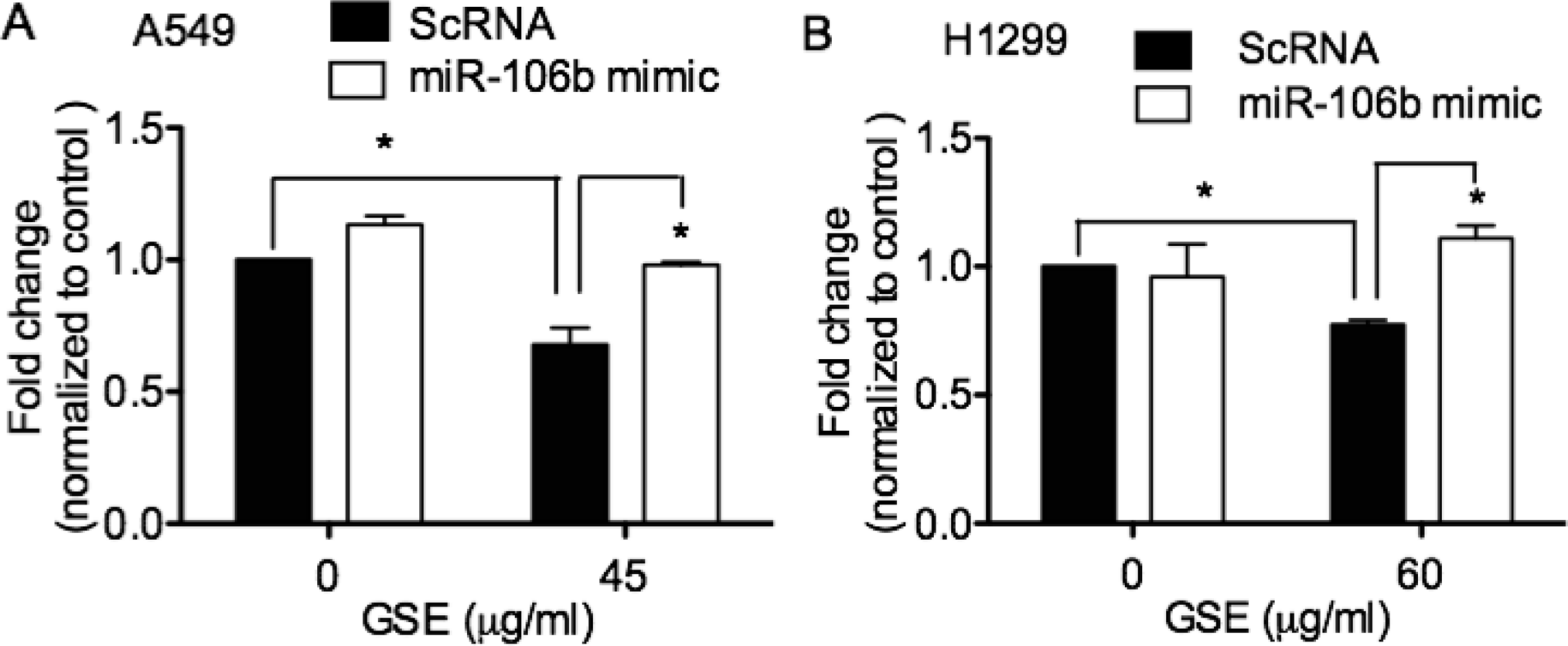
GSE induced anti-proliferative effects in lung neoplastic cells via down-regulation of miR-106b Quantification of cell proliferation in conditioned A549 and H1299 cells was assessed by MTT assay. Overnight GSE treatment significantly reduced (**A**) A549 and (**B**) H1299 cell proliferation, but not in NHBE cells, data not shown [9]. The GSE-induced anti-proliferative effects in lung neoplastic cells was abrogated by transfection of miR-106b mimics. Control represented cells treated with scrambled oligonucleotides, ScRNA. Columns, mean; bars, SD. ^*^P < 0.05.

To ascertain the role of miR-106b in mediating the anti-neoplastic property of GSE, we evaluated the ability of miR-106b mimic transfection in reversing the anti-proliferative effects of GSE in A549 and H1299 cells. MiR-106b mimics significantly abrogated the GSE mediated anti-proliferative effects in these lung cancer cells (Figure 2A and 2B).

### GSE increased *CDKN1A*/P21 in lung cancer cells via down-regulation of miR-106b

**Figure 3:**
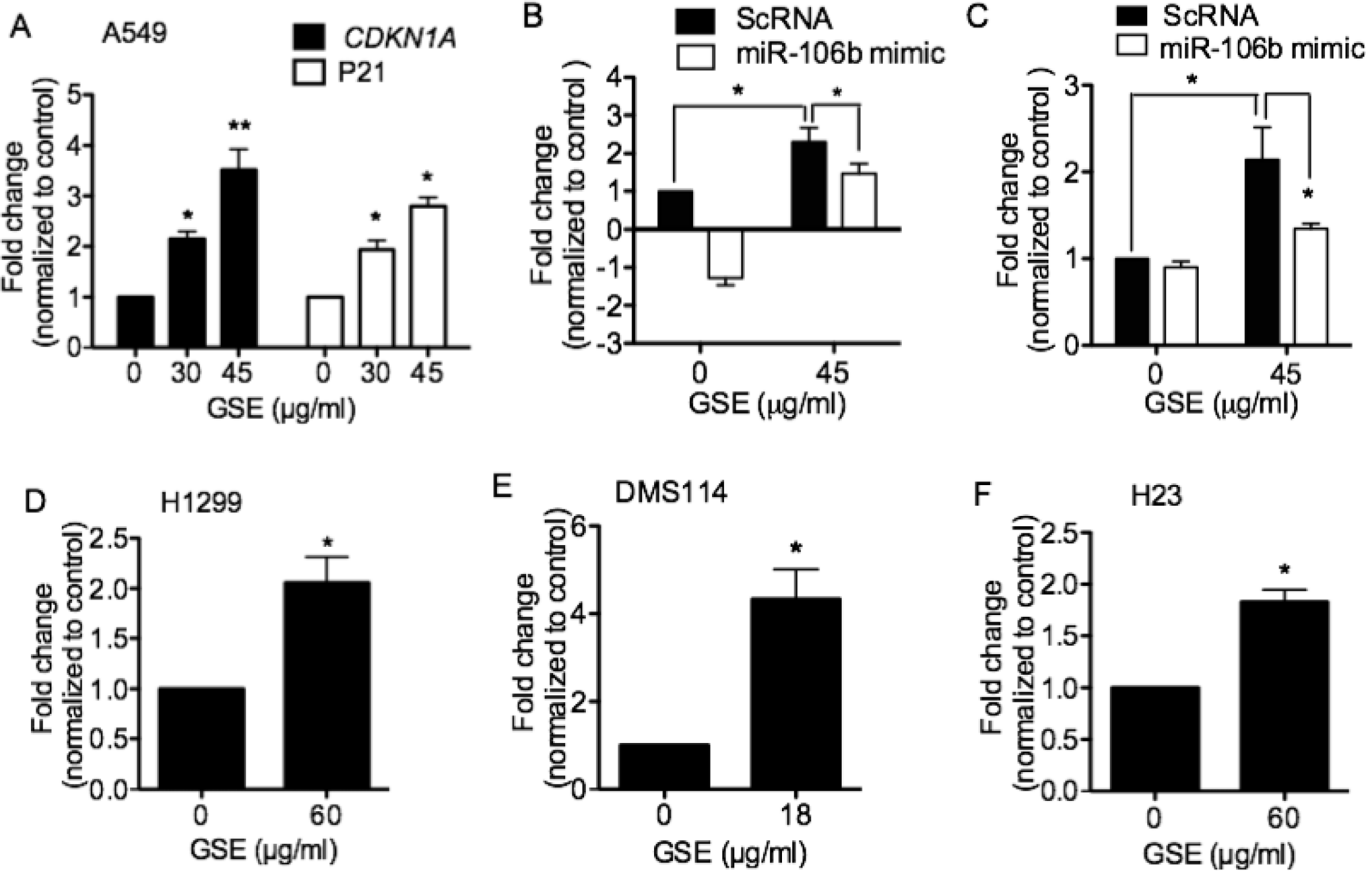
GSE significantly increased (**A**) mRNA expressions of *CDKN1A* and p21 protein production, and miR-106 mimic abrogated such increases in (**B**) *CDKN1A* mRNA expression and (**C**) p21 protein production in A549 cells. GSE also significantly increased *CDKN1A* mRNA expression in (**D**) H1299, (**E**) DMS114, and (**F**) H23 cells. Columns, mean; bars, SD (*n* = 3). ^*^P < 0.05, ^**^P < 0.01).

We then evaluated the effects of GSE treatment on predicted targets of miR-106b that are known to play a role in cell proliferation, including CDKN1A or p21. P21 is a potent cyclin-dependent kinase inhibitor that functions as an inhibitor of cell cycle progression. GSE significantly and dose-dependently increased both *CDKN1A* mRNA expressions and p21 production in A549 cells (Figure 3A). Transfection of miR-106b mimics partially abrogated the GSE-mediated up-regulation of *CDKN1A* mRNA expression (Figure 3B) and p21 production (Figure 3C). Additionally, GSE significantly increased *CDKN1A* mRNA expression in H1299 (Figure 3D), DMS114 (Figure 3E) and H23 (Figure 3F) cells. However, GSE did not increase *CDKN1A* mRNA expression in H520 cells (data not shown).

### GSE induced anti-invasive effects in lung neoplastic cells via down-regulation of miR-106b, which was abrogated by transfection of miR-106b mimic

**Figure 4:**
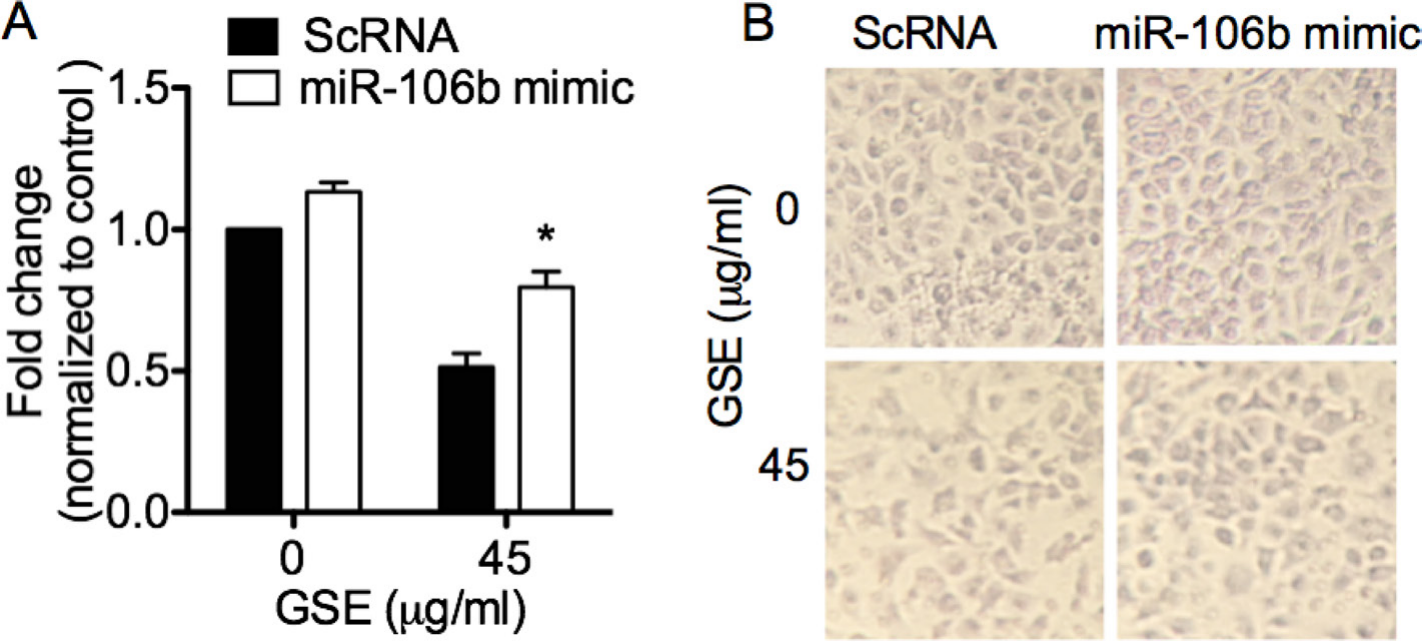
GSE induced anti-invasive effects in lung neoplastic cells via down-regulation of miR-106b, which was abrogated by transfection of miR-106b mimic GSE treatment significantly reduced the invasion of A549 cells through the 8 µM pore size co-culture inserts coated with matrigel as extracellular matrix (ECM), and transfection of miR-106b mimic significantly abrogated the GSE-mediated anti-invasive effects in A549 cells (**A**). (**B**) Representative photomicrographs of conditioned A549 cells on the bottom side of the inserts that have invaded through the matrigel. Control represented cells treated with scrambled oligonucleotides, ScRNA. Columns, mean; bars, SD. ^*^P < 0.05.

To ascertain the role of miR-106b in mediating the anti-invasive property of GSE, we evaluated the ability of miR-106b mimic transfection in reversing the reduction of A549 cell invasion by GSE. MiR-106b mimics significantly abrogated the GSE-mediated anti-invasive effects in A549 cells (Figure 4A and 4B).

### LP treatment significantly down-regulated *MIR106B* mRNA, miR-106b, and up-regulated *CDKN1A* mRNA expressions in human lung tumor xenografts

**Figure 5:**
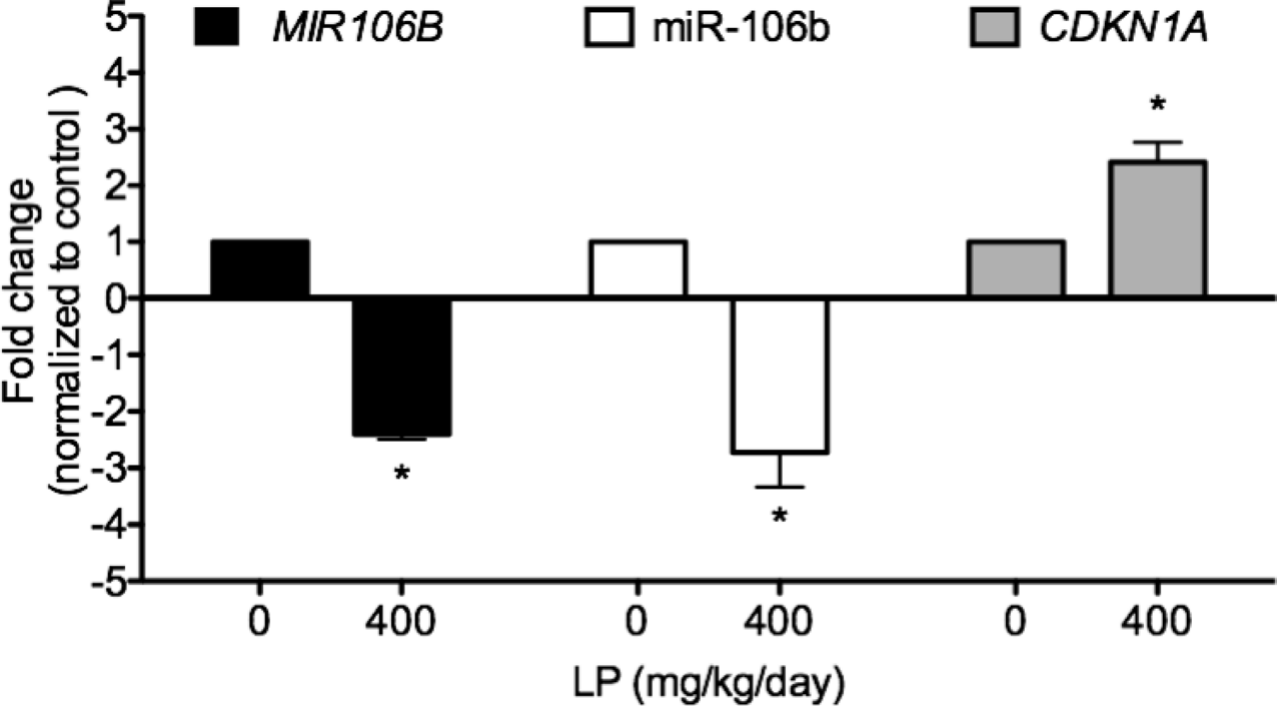
LP treatment significantly down-regulated *MIR106B* mRNA, miR-106b, and upregulated *CDKN1A* mRNA expressions in human lung tumor xenografts To confirm that the *in vitro* effects of GSE do occur *in vivo*, we assessed and compared the expressions of *MIR106B,* miR-106b, and *CDKN1A* in lung tumor xenografts with or without LP treatment using qPCR. Eight weeks of oral LP treatment (water vs. LP of 400 mg/kg/day containing 112 mg/kg/day of GSE) via gavage, significantly down-regulated expressions of miR-106 and mRNA of its precursor gene *MIR106B*, and up-regulated mRNA expression of *CDKN1A*. Columns, mean; bars, SD (*n* = 5). ^*^P < 0.05.

To confirm that the *in vitro* effects of GSE do occur *in vivo*, we assessed and compared the expressions of precursor *MIR106B* mRNA, miR-106b, and *CDKN1A* mRNA in lung tumor xenografts with or without LP treatment. Oral LP treatment significantly down-regulated expressions of miR-106b, *MIR106B* mRNA, and up-regulated mRNA expression of *CDKN1A* (Figure 5).

### Plasma GSE and metabolites as markers of bioavailability for LP treatment

**Figure 6:**
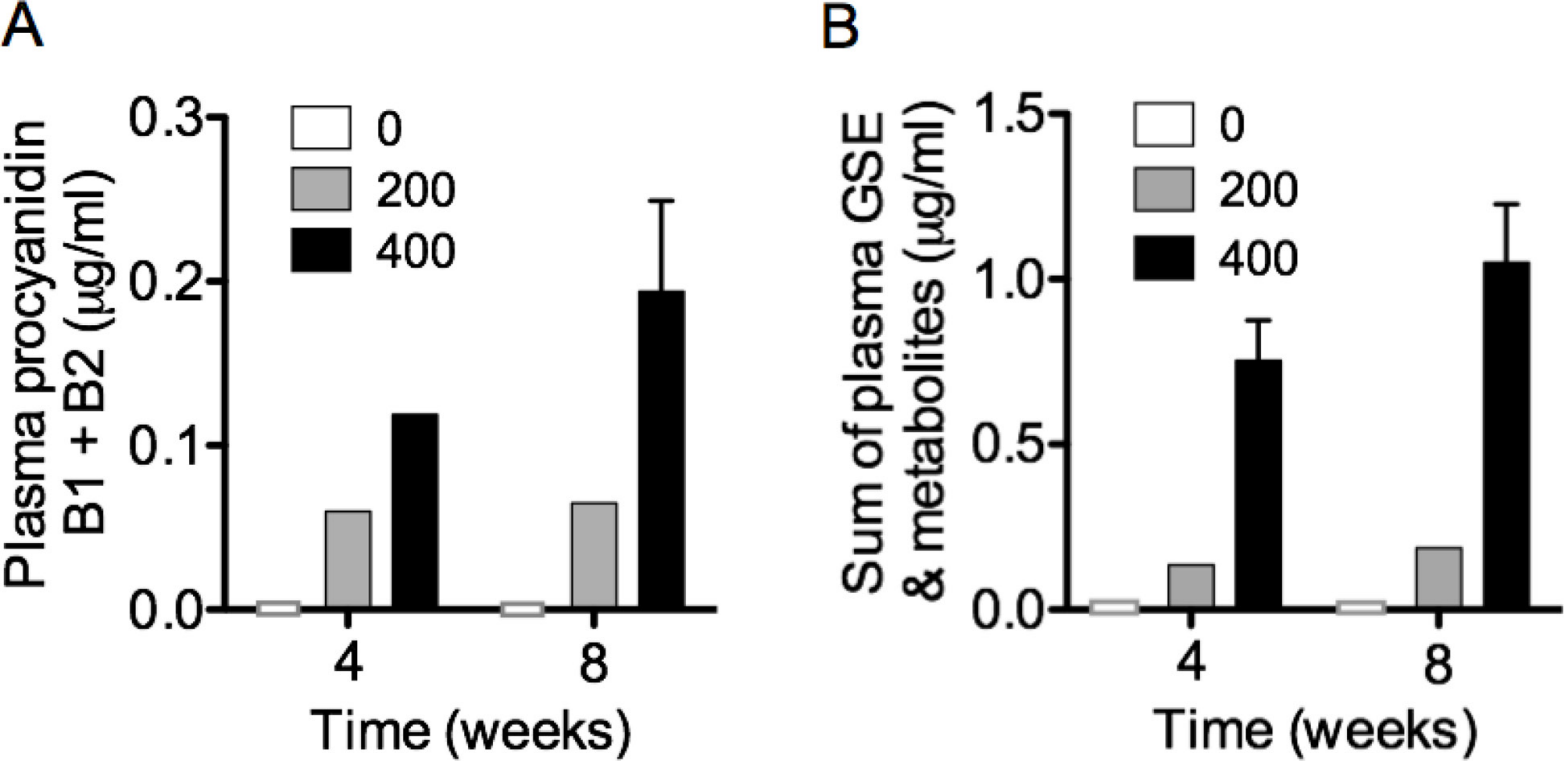
Plasma GSE and metabolites were measured as markers of bioavailability for LP treatment The systemic bioavailability of oral LP, including procyanidins B1 and B2, catechin, epicatechin, procyanidin gallate, epicatechin gallate, methyl catechin, methyl-epicatechin and methyl-epicatechin gallate, were measured in mouse plasma obtained between 60–90 minutes after gavage of LP at week 4 and 8 of treatment. The plasma concentrations of (**A**) procyanidins B1 + B2 and (**B**) sum of GSE + metabolites appeared to be sustained or increased over time with continued dosing.

To determine the systemic bioavailability of oral LP, levels of GSE and metabolites including procyandiins B1 and B2, catechin, epicatechin, procyanidin gallate, epicatechin gallate, methyl catechin, methyl-epicatechin and methyl-epicatechin gallate, were measured in mouse plasma obtained between 60–90 minutes after gavage of LP at week 4 and 8 of treatment. The plasma concentrations of procyanidins (Figure 6A) and sum of GSE + metabolites (Figure 6B) appeared to be sustained or increased over time with continued dosing.

### Lung homogenates from LP treated nude mice significantly reduced proliferations of various lung cancer cell types

**Figure 7:**
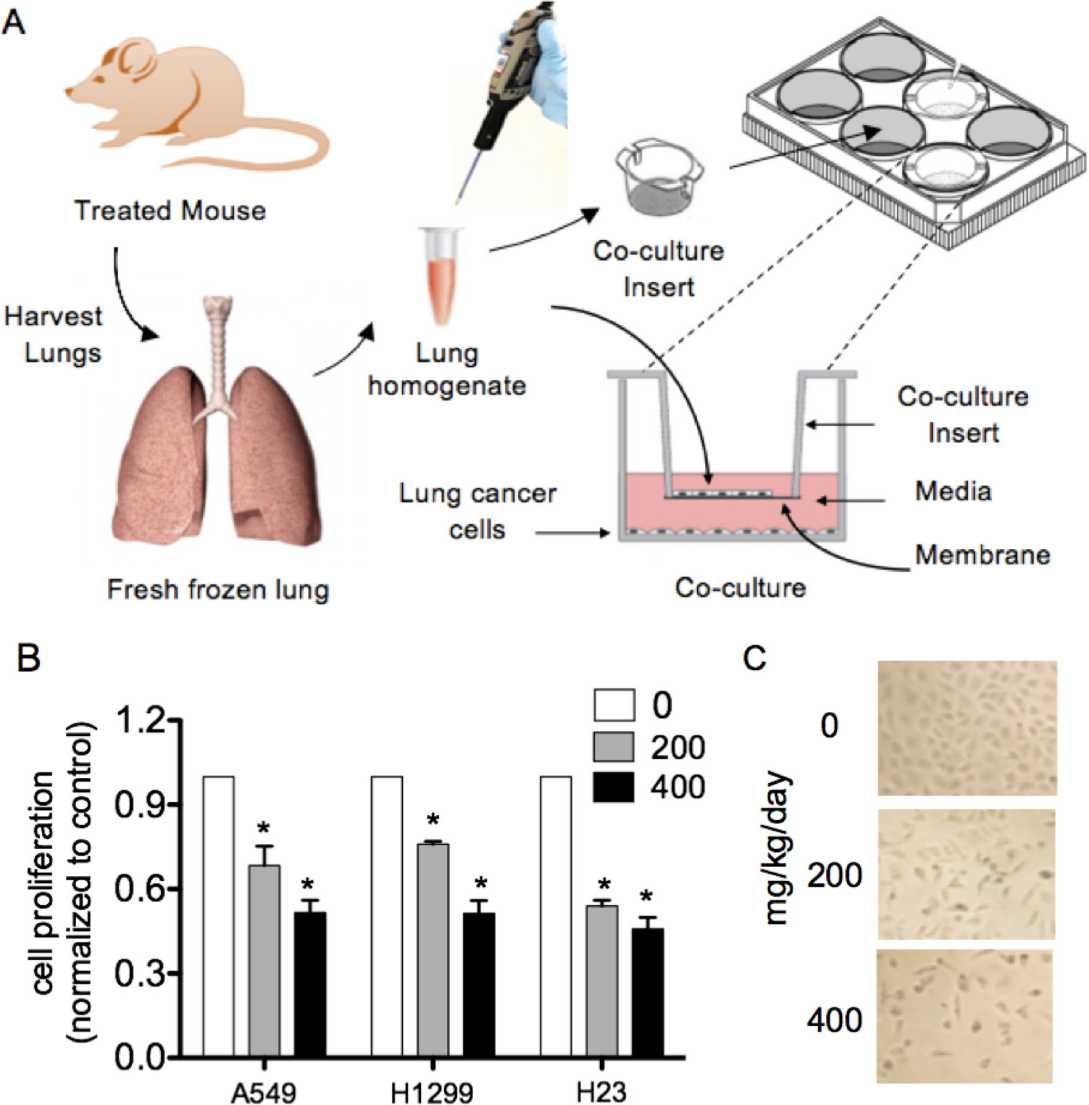
Lung homogenates from LP treated nude mice significantly reduced lung cancer cell proliferation in comparison to water-treated mice Fresh, snap frozen whole lung homogenates from nude mice in our LP efficacy study were used. We placed 25 μl of homogenate on 0.4 µM pore size membrane culture insert for 12 well plates, then combined the inserts in 12 well culture plates with A549, H1299 or H23 cells grown overnight on the bottom wells (cells at exponential growth phase, approximately 60–70% confluence). The Co-cultures were then incubated overnight. (**A**) Co-culture schema. (**B**) Co-culture with lung homogenates from LP treated mice significantly and dose-dependently reduced lung neoplastic cell proliferations. (**C**) Representative photomicrographs of A549 cells co-cultured with lung homogenate. Columns, mean; bars, SD (*n* = 3). ^*^, P < 0.05.

To demonstrate the feasibility of a co-culture system as a surrogate bioassay to assess the bioavailability and bioactivity of an orally administered natural agent in the lungs, we developed a method using fresh, snap frozen whole lung homogenates (standardized by wt./volume) from nude mice treated with LP in our efficacy study. We placed lung homogenate on culture insert for 12-well plates, then combined the inserts in 12-well culture plates pre-plated with A549, H1299 or H23 cells grown overnight on the bottom wells. The co-cultures were then incubated overnight (Figure 7A). Co-culture with lung homogenates from LP treated mice significantly and dose-dependently reduced lung neoplastic cell proliferations (Figure 7B and 7C).

## DISCUSSION

In this study, we report for the first time, the roles of miR-106b, and its downstream target CDKN1A or p21, in mediating the anti-neoplastic properties of GSE against NSCLC and SCLC. We also demonstrate the utility of a novel fresh frozen whole lung homogenate co-culture system for assessing bioavailability and antitumor bioactivity of orally administered LP to the target organ of interest. We further report the plasma levels of GSE and the sum of GSE and metabolites levels in nude mice from our modified PK study with oral administration of LP, the concentrations of which are substantially lower than the IC_50_ required to achieve similar degree of tumor growth inhibition *in vitro.*

Aberrant expression of miRNA has been implicated as a driver of tumorigenesis/promotion, including classical cancer pathways such as increases in cell proliferation, angiogenesis and resistance to apoptosis. MiR-106b is one of these oncomirs that has been reported to play a role in tumors of many organs including lung [14–20]. Down-regulation of miRNA-106b by GSE has been shown to inhibit growth of melanoma cells by promoting G1-phase cell cycle arrest and reactivation of p21 protein [21]. In our study, we also show that GSE down-regulates miR-106b, which in turn up-regulates *CDKN1A* gene mRNA expression and its respective protein p21 production in our lung cancer models. Furthermore, we demonstrate that GSE down-regulates the mRNA expression of *MIR106B* precursor gene. We previously reported that GSE down-regulated the miR-17-92 cluster host gene (*MIR17HG*) and miR-19a/b in various NSCLC (A549, H520, H1299) and bronchial premalignant cancer cells, leading to up-regulations of their downstream targets - tumor suppressors PTEN and IGF2R [8]. As the *MIR106B* gene is a paralogue of mir-17-92 cluster, it is conceivable that GSE down-regulates miR-106b via similar molecular mechanisms at the level of miRNA precursor gene transcription in lung tumors. The ability of GSE in modulating miR-106b and its downstream targets provides further evidence on its multi-faceted antineoplastic properties against lung cancer. However, it is noteworthy that GSE did not significantly decrease *MIR106B* nor miR-106b expression in H520, a squamous cell carcinoma cell line, in contrast to what was observed with miR-19a/b. Yet from a phenotypic standpoint (anti-proliferative and induction of apoptosis), GSE was very effective against H520. This finding suggests that the differential effects of GSE on oncomirs expressions are likely cell type specific, may potentially involve differential regulation of specific RNA processing enzymes in different lung cancer cell types that contribute to the heterogeneity of lung cancer biology. The precise mechanisms accounting for such variations among different cell types may have important clinical implications toward the development of precision medicine and identification of novel molecular targets for lung cancer. Further investigation is warranted but is beyond the scope of the present study and remains to be elucidated.

CDKN1A or p21 is a potent cyclin-dependent kinase inhibitor capable of inhibiting all cyclin/CDK complexes, although it is primarily associated with inhibition of CDK2. P21 promotes cell cycle arrest in response to many stimuli and is well positioned to function as both a sensor and an effector of multiple anti-proliferative signals [22]. The P21 protein is encoded by the *CDKN1A* gene located on chromosome 6 (6p21.2) in humans [23]. P21 represents a major target of p53 activity and is associated with linking DNA damage to cell cycle arrest. This protein can interact with proliferating cell nuclear antigen, a DNA polymerase accessory factor, and plays a regulatory role in S phase DNA replication and DNA damage repair. This is consistent with our findings that transfection of miR-106b mimics abrogated the antiproliferative effect of GSE in lung neoplastic cells. In addition, p21 has been shown to be specifically cleaved by caspase 3-like caspases, which leads to a dramatic activation of CDK2, and may be instrumental in the execution of apoptosis following caspase activation. We previously reported that GSE induced apoptosis in NSCLC cells via the activation of caspase 3 [9]. Conceivably, the activation of caspase 3 is mediated, in part, through an increase in p21 production from the down-regulation of miR-106b.

In addition to mediating the anti-proliferative effects of GSE, we found that miR-106b was involved in the anti-invasive effects of GSE in lung cancer, another important hallmark of the malignant phenotype. In a recent report, the p53/p21 complex rather than p53 itself regulates cell invasion and death by targeting Bcl-2 proteins [24], suggesting that the GSE-induced down-regulation of miR-106b likely reduce lung cancer cell invasiveness, in part, though the increase in P21 production.

Because the absorption of GSE is affected by molecular wt. and the variable compositions of GSE polyphenols in different products on the market also contribute to low and erratic bioavailability [25–26], we selected LP, a GSE preparation standardized to smaller size oligomeric procyanidins (OPC) and complexed with soy phospholipids into phytosomes (1:2.6 w/w) to improve bioavailability, as the agent of choice in our preclinical study. A modified PK study was conducted to ascertain the bioavailability of GSE and metabolites following oral administration of LP in nude mice. Procyanidins are metabolized to catechin, epicatechin, epicatechin gallate, methyl catechin, methyl-epicatechin and methyl-epicatechin gallate. Even with the assumption that all these polyphenols are biologically active, the sum of these polyphenols in the plasma is still substantially lower (more than 1 order of magnitude) than the *in vitro* dose required to achieve IC_50_ on tumor cell proliferation. Once again, our findings confirm the limitations of *in vitro* studies and the importance of *in vivo* PK studies in defining physiologic relevant dosing in reference to bioactivity and efficacy of an agent. Our modified PK study also demonstrated that chronic administration of LP did not induce its auto-metabolism, as the procyanidins and metabolites levels appear to be sustained and increase over time.

To further correlate the bioavailability of LP to bioactivity at our target organ of interest, and to demonstrate that oral administration of LP can reach and exert antineoplastic effects in the lungs, we developed a co-culture system using fresh frozen lung homogenate obtained from control and LP-treated mice. As anticipated, lung homogenate from mice gavaged with water did not have any effect on lung cancer cell proliferation, whereas homogenates from LP-treated mice significantly and dose-dependently reduced cell proliferation in all lung cancer cell lines tested. This novel co-culture system is easy to set up; it not only allows rapid assessment of bioavailability/bioactivity of LP in the lungs, but also presents a roadmap for future applications in preclinical drug development studies for other organ systems beyond the lungs, as similar co-culture approach can easily be adapted to assess bioactivity of treatment in other organs of interests.

Lung cancer is the leading cause of cancer death in the world. Despite advancements in anti-cancer treatment, including targeted molecular therapy, the 5-year survival for both NSCLC and SCLC remains dismal [27–32]. The lack of effective therapy provides the impetus to search for alternative, safe and efficacious antineoplastic agents against lung cancer, to impede the driving force of cancerization, and prevent lung cancer development in at-risk individuals. In this study, we demonstrate novel GSE mediated anti-neoplastic mechanisms involving modulation of oncomir miR-106b, its molecular target p21, and correlated these findings to the *in vivo* efficacy of LP, against lung cancer. Modulations of these signaling pathways may be useful as SEBM and predictor of response in future clinical trials. We also report the systemic bioavailability of oral LP, and bioactivity at the target organ using a novel lung homogenate co-culture system. In view of the complexity and heterogeneity of lung tumor biology in humans and the safety profile of the natural agent, it is conceivable that GSE may be used in combination with chemotherapy and/or radiation, or as adjuvant or neoadjuvant treatments to enhance overall efficacy of antineoplastic regimen against lung cancer. Our findings provide important rationales to further investigate the potential of GSE against both NSCLC and SCLC.

## MATERIALS AND METHODS

### Cell culture

As models to evaluate the anti-neoplastic effect of GSE against lung cancer, the human NSCLC lines, A549 (Adenocarcinoma), H520 (squamous cell carcinoma), H1299 (Metastatic NSCLC), DMS114 (small cell lung cancer), H23 (adenocarcinoma) (ATCC; Manassas, VA, USA), and normal human bronchial epithelial cells (NHBE) (Lonza Inc. Allendale, NJ, USA) were studied *in vitro*. Experiments involving all commercial cell lines were initiated within 6 months of purchase. Cell lines were not further authenticated. ATCC uses Short Tandem Repeat profiling for cell line authentication. Cells were maintained as monolayers in an atmosphere of 5% CO_2_ in air at 37° C in 25-cm^2^ tissue culture flasks containing 5.0 ml of RPMI-1640 medium supplemented with 10% FBS, 100 units/ml of penicillin, 0.1 mg/ml of streptomycin, and 2 mM of glutamine (JRH Biosciences; Lenexa, KS, USA) for A549; and RPMI-1640 medium with 2 mM L-glutamine adjusted to contain 1.5 g/L sodium bicarbonate, 4.5 g/L glucose, 10 mM HEPES, and 1.0 mM sodium pyruvate, 90%; fetal bovine serum, 10% for H1299 and H520 cells. Aliquots of 0.1 × 10^6^ A549 or H520 cells were incubated at 37° C for 2 h. Varying doses of GSE (0, 18, 30, 36, 45, and 60 μg/ml) were added and the cells were incubated at 37° C for 18–44 h. The dose range of GSE was chosen based on considerations from prior published studies [6–8]. Primary normal human bronchial epithelial (NHBE) cells was used as control. NHBE cells were maintained according to the manufacturer’s instructions. Aliquots of 0.5 × 10^6^ of NHBE were plated in wells and incubated at 37° C for 18–44 h. For MTT assays, cells were plated at concentration of 6–8 × 10^3^ cells/well in 96 well plates, conditioned and cultured for 18–44 h.

All conditioned culture supernatants and total RNA were harvested with cell lysates stored at –80° C until analysis, when applicable. Samples of mRNA were collected using the miRNeasy Mini Kit (Qiagen Inc, Valencia, CA, USA), per the manufacturer’s instructions.

### GSE preparations

Standardized GSE (90% procyanidins, which were members of the proanthocyanidins class of flavonoids), was purchased from Organic Herb Inc., China. Stock solutions of GSE were made by dissolving the extract with deionized water. Aliquots of the stock were stored at –80° C and used only once for each set of experiments. For oral gavage, suspensions of varying doses of LP (Indena Inc., Milan, Italy), comprised of standardized oligomeric procyanidins complexed with soy phospholipid (1:2.6 w/w), were freshly prepared daily in deionized water for each treatment group just prior to gavage.

### Animals and tumor xenograft assay

Female athymic nude mice (8–9 weeks old) were xenografted, and treated with LP as previously described [8]. Briefly, exponentially growing A549 cells were mixed at a 1:1 ratio with Matrigel (Trevigen Inc. Gaithersburg, MD, USA), and a 100 μL suspension containing 1.2 × 10^6^ cells was injected subcutaneously in the right flank of each mouse. Mice were randomly divided into 4 treatment groups (*n* = 9 per group), and gavaged every morning with varying doses of LP (0, 200, 300 and 400 mg/kg). Clinical scoring including body wt, signs of illness or suffering were assessed daily and tumor growth was regularly monitored. Tumor size was determined using the ellipsoid volume formula (π/6 × L × W × H) [33]. The experiment was terminated at 56 days after tumor cell inoculation following the guidelines of Institutional Animal Care and Use Committee at the New Mexico VA Health Care System. Plasma, lungs and tumors (1/2 fresh frozen, 1/2 formalin fixed and paraffin embedded FFPE) were harvested at various time points for biomarker determination.

### Quantification of cell proliferation: MTT assay

To quantify cellular proliferation in conditioned cells, The MTT Cell Proliferation Assay (ATCC; Manassas, VA, USA) was used according to the manufacturer’s instructions.

### Cell invasion assay

Conditioned cells were plated in serum free medium (1.5 × 10^5^ cells/ml), with 300 μl plated per 8.0 µM pore size co-culture inserts for 24 well plate (Corning biocoat, Fisher Scientific) that were precoated with matrigel, then combined with the 24 well culture plate containing medium with 10% FBS in the bottom wells. After 24–48 h incubation, cells remaining on the top layer of the culture insert were removed by cotton swabs, cells that have migrated/invaded through the matrigel were fixed and stained with toluidine blue and counted in at least 5 randomly selected, fields.

### Real time (q) PCR for quantification of miRNA and mRNA expression

The total RNA isolated using miRNeasy Mini kit was converted to first strand cDNA via universal tailing and reverse transcription. The cDNA template was mixed with qPCR Master Mix and aliquoted into each well of the 96-well plate containing an array of pre-dispensed miRNA-specific primer sets (MAH-100, SA Bioscience; Fredrick, MD, USA). QPCR was performed on the Bio-Rad MyiQ cycler (Bio-Rad; Hercules, CA, USA). Following identification of miRNA of interest, further validation using qPCR with specific miR-106b primers was performed, per manufacturer’s instructions. The qPCR reactions for the *CDKN1A* gene and the *MIR106B* gene were performed using reagents, specific primers from SA Bioscience per the manufacturer’s instructions. Any C_t_ greater than 35 was considered a negative call. The values were first normalized to beta-actin, then to control, using ΔΔC_t_ based fold-change calculations from raw threshold cycle (C_t_) data. Data are depicted in fold changes normalized to control. Negative fold change represents down-regulation; a reduction of 50% or 75% from control (untreated cells) is equivalent to -2 or -3 fold changes, respectively.

### MiRNA *in situ* hybridization assay

*In situ* hybridization (ISH) of miR-106b was performed using the QuantiGene^**®**^ ViewRNA miRNA ISH cell assay kit (Affymetrix Panomics, Santa Clara, CA, USA). Briefly, 8 × 10^3^ cells/well were plated in a 96 well plate precoated with Poly-L-Lysine. After 2 h adherence, cells were conditioned with varying doses of GSE overnight, then fixed in 4% formaldehyde, cross linked with EDC, permeablized with detergent and digested with protease and then hybridized to target probes, followed by amplification and detection steps as per manufacturer’s instruction.

### MiRNA mimic transfection

Transfections of miR-106b mimic into lung neoplastic cells were achieved using miRNA specific mimic, and transfecting reagents according to the manufacturer’s instructions (Qiagen Inc. Valencia, CA, USA). Briefly, 6–8 × 10^3^ of cells in 150 μl of cells were aliquoted into 96-well plates. After 1 h of adherence, 50 μl of specific miRNA transfection complexes were gently added to the cells with gentle swirling. The cells were incubated for 4 h, then conditioned overnight with GSE, followed by the MTT assay. Cell conditions were scaled up 10 fold in 12 well plates for total RNA harvest.

### P21 ELISA

Human Total p21 ELISA kit was used to quantify p21 protein in conditioned cell lysates per the manufacturer’s instructions (R & D Systems, Minneapolis, MN, USA). P21 levels from each sample were referenced to the concentrations of total protein. Total protein levels were determined using the Pierce BCA Protein Assay kit per the manufacturer’s instruction (ThermoScientific, Rockford, IL, USA).

### Lung homogenate co-culture

Snap fresh frozen whole lungs harvested from nude mice treated with water and LP (200 or 400 mg/kg/day) in our maximum tolerated dose (MTD) study were homogenized in RPMI medium (40 mg/μl), using a handheld Homogenizer (TissueRuptor, Qiagen, Valencia, CA, USA). Twenty-five μl of lung homogenate per condition was added onto a 0.4 μm co-culture insert, then the inserts were combined in a 12-well culture plate pre-plated with 0.6 × 10^5^ cells/ml, 1 ml/well of A549 or H23 cells, or 0.4 × 10^5^ cells/ml, 1 ml/well of H1299 cells grown overnight on the bottom wells. The co-cultures were then incubated overnight. The effects of GSE-treated lung homogenate on lung cancer cell proliferation were determined.

### Measurement of plasma procyanidins and metabolites

Procyanidins were extracted from mouse plasma (0.2 mL) by incubating samples with 0.3 mL of enzyme solution (1000 U β-glucuronidase with 40 U sulfatase activity in 0.5 M NaH_2_PO_4_ pH 5.0 containing 2% ascorbic acid) for 45 minutes at 37° C. Following incubation, samples were extracted three times with ethyl acetate/2% ascorbic acid. Combined ethyl acetate extracts were dried, and residue was resolublized in 100 μL 50% aqueous methanol prior to analysis using LC/MS. Briefly, separation was performed on a Surveyor HPLC system equipped with a diode array detector (Thermo Finnigan, San Jose, CA, USA) using a Zorbax SB-C18 column (4.6 × 150 mm, 3.5 μm; Agilent, Santa Clara, CA, USA). A binary mobile phase consisting of solvent systems A and B was used in gradient elution where A was 1% acetic acid (v/v) in ddH_2_O and B was acetonitrile. Mobile phase flow rate was 0.75 mL/min with a T splitter to make 0.25 mL/min flow to MS. Initial conditions was set at 98:2 A:B with a linear gradient to 75:25 from 0 to 25 min, and to 60:40 from 25 to 32 min. Following separation, the column effluent was introduced by negative mode electrospray ionization (ESI) into an LCQ Advantage Ion Trap Mass Spectrometer. ESI capillary voltage was -4.0 kV, capillary temperature was 275° C, sheath gas flow was set at 40, and the normalized collision energy was set at 45%. Spectroscopic (UV at 280 nm) and MS/MS data (577/425) was collected and analyzed using Xcalibur software (Thermo Finnigan). Concentrations of plasma procyanidin B1 and B2 were determined by internal calibration and ethyl gallate was used as an internal standard.

### Statistical analysis

Data were expressed as the mean ± SD in all circumstances where mean values are compared. Data were analyzed by paired Student’s *t* test and/or ANOVA. Batch analyses were performed for each comparison group to eliminate interassay variability. Differences are considered significant when *p* < 0.05.

## Abbreviations

GSE: grape seed procyanidin extract
CDKN1A: cyclin-dependent kinase inhibitor 1A
LP: leucoselect phytosome
PK: pharmacokinetics
NSCLC: non-small cell lung cancer
SCLC: Small cell lung cancer
SEBM: surrogate endpoint biomarkers
ScRNA: scrambled oligonucleotides
